# Editorial: Grievance-fueled violence: conceptual and empirical developments

**DOI:** 10.3389/fpsyg.2023.1177705

**Published:** 2023-05-11

**Authors:** Emily Corner, Troy McEwan, Caroline Logan

**Affiliations:** ^1^Centre for Social Research and Methods, Australian National University, Canberra, ACT, Australia; ^2^Centre for Forensic Behavioural Science, Swinburne University of Technology, Alphington, VIC, Australia; ^3^Greater Manchester Mental Health National Health Service Foundation Trust and School of Health Sciences, University of Manchester, Manchester, United Kingdom

**Keywords:** grievance, violence, mental health, security, offending

Acts of extreme or mass violence perpetrated by lone individuals have become increasingly common in liberal democracies over the past 20 years. Despite an enduring interest in violent extremism, there remains a lack of consistency in the demographic, ideological, and psychological profiles of the perpetrators of these incidents as a whole. Some of these acts have been described as politically motivated terrorism, whilst others have been attributed to mental illness or criminal intent. However, there is an increasingly common view that the distinction between political ideology, criminal intent, and personal motivations is often blurred (Böckler et al., 2018; Clemmow et al., 2022) and that the violence carried out by these individuals is better understood using the broader concept of grievance-fueled violence. Grievance-fueled violence is not just a theoretical construct. Multi-agency teams of specialist police and mental health clinicians have been established in Australia, the Netherlands, New Zealand, and the United Kingdom to help mitigate the threat presented by lone individuals with complex grievances (Pathé et al., 2018).

A major focus of each article in this issue is the potential for benefits in the conceptual shift from siloed areas of research focusing on terrorism, mass murder, fixation, hate crimes, fatal family violence, and incel-related violence to the unifying concept of grievance-fueled violence. For example, Ebbrecht and Lindekilde argue through their case study of the Aarhus University shooting that the nuances in offender motivation can be lost when such violent acts are classified in traditional typological frameworks. Sizoo et al. lend further support, noting that although the current explanatory frameworks were conceptualized to understand the specific forms of violence, new acts of violence by lone offenders suggest that the current frameworks may not be completely satisfactory. And James et al. and Cooper et al. contend that, given this, the adoption of a grievance-fueled violence framework would allow the application of insights gained in the study of one form of offense to be applied to others.

Current research on this topic is characterized by its diversity. The papers in this topic explore a range of offense types and apply the concept of grievance-fueled violence to better understand the underlying motivations of offenders. Binder and Kenyon situate online radicalization to terrorism within the wider context of attitudes and beliefs that develop from grievance, arguing that the evolving threat environment may be better understood under the concept of grievance-fueled violence. Alberda et al. emphasize how the concept of grievance may offer insight into risk and protective factors found across terrorist offenders. Higgs et al. critically examine the scope of grievance in the context of sexual violence, arguing that the examination of sexually harmful offenders may offer deeper insight into the development of grievance-fueled violence.

Collectively, the papers in this Research Topic demonstrate the potential for applying the concept of grievance-fueled violence to acts of violence by lone offenders. However, together they note that theoretical interrogation and development are required given the novelty of the concept (Brooks and Barry-Walsh; Higgs et al.; Sizoo et al.). Brooks and Barry-Walsh propose that, though the concept of grievance-fueled violence is blurred and lacks definitive rules and boundaries, it can nonetheless be used to help guide the conceptual development of our understanding of a diverse group of offenders who enact targeted violence. To aid this development, Sizoo et al. propose the application of enactivism, highlighting that such an approach understands grievance as a dynamic, interpersonal, and context-sensitive construct.

The major limitation in this research area currently is the lack of empirical interrogation (Pathé et al., 2018). This Research Topic makes a concerted effort to resolve this. Indeed, half of the papers focus on novel empirical research. Ebbrect and Lindekilde critically analyse existing open- and closed-source data, including police reports and correspondence from the offender, on the Aarhus University shooting. They conclude that the development of the offender's grievance was complex and characterized by resentment regarding multiple issues, social deprivation, interpersonal rejection, and rumination on violence in the context of the offender's personality structure and poor mental health. James et al. interrogate 126 cases of concerning communications and approaches to public figures. They highlight the importance of scrutinizing motivations, noting that those classified as holding a motivation of grievance showed significant associations with approach behavior in the overall number of threats, number of direct threats, number of condition threats, and number of categories of threats used. However, the authors note that these effects disappeared when motivation is not considered in the analysis. Alberda et al. explore differences between risk and risk mitigating factors between terrorist offenders who commit homicide and other terrorist offenders. They highlight that terrorists who commit homicide are more likely to present with some form of grievance. Cooper et al. present a scoping review and analysis comparing the characteristics and motivations of perpetrators of fatal family violence and lone actor grievance-fueled homicide offenders. The authors conclude that over half of the fatal family violence cases shared distinct similarities with those who committed lone actor grievance-fueled homicide in other contexts.

In short, the research presented here offers promise for the development of the concept of grievance-fueled violence. We hope that the papers in the Research Topic will stimulate further research in this field to help mature the current theoretical constructs, in turn helping to refine both the empirical focus and the ongoing development of mitigation and prevention efforts.

## Author contributions

EC, TM, and CL conceived and shared the task of editing the Research Topic. EC wrote the first draft of the editorial, to which TM and CL then contributed. All authors contributed to the article and approved the submitted version.
